# Evaluation of a novel *Aspergillus* IgG lateral flow assay for the diagnosis of non-neutropenic patients with acute and subacute invasive aspergillosis

**DOI:** 10.3389/fcimb.2025.1599425

**Published:** 2025-06-20

**Authors:** Yajie Lu, Huanhuan Zhong, Yujie Wang, Chao Sun, Yuanyuan Li, Yuchen Cai, Xiaomin Cai, Jiamei Wang, Jinjin Zhong, Xin Su

**Affiliations:** ^1^ Department of Respiratory and Critical Care Medicine, Nanjing Drum Tower Hospital, Affiliated Hospital of Medical School, Nanjing University, Nanjing, China; ^2^ Department of Respiratory and Critical Care Medicine, The Second Affiliated Hospital of Soochow University, Suzhou, China; ^3^ Department of Respiratory and Critical Care Medicine, Nanjing Jinling Hospital, Affiliated Hospital of Medical School, Nanjing University, Nanjing, China; ^4^ Department of Respiratory and Critical Care Medicine, Nanjing Jinling Hospital, Medical School, Nanjing Medical University, Nanjing, China

**Keywords:** acute invasive aspergillosis, subacute invasive aspergillosis, non-neutropenic patients, *Aspergillus* IgG lateral flow assay, diagnosis

## Abstract

**Purpose:**

This study aimed to assess a novel lateral flow assay (LFA) for *Aspergillus* IgG detection in patients with non-neutropenic invasive aspergillosis (IA).

**Methods:**

*Aspergillus* IgG LFA and enzyme-linked immunosorbent assay (ELISA) were performed in non-neutropenic IA patients and control group (proven community acquired pneumonia and healthy persons), respectively. The diagnostic performance of *Aspergillus* IgG LFA for IA was evaluated and compared with ELISA method.

**Results:**

33 cases of acute IA, 30 cases of subacute IA and 80 controls were enrolled in this study. The level of plasma *Aspergillus* IgG LFA in the IA group was significantly higher than that in the control group (190.5 AU/mL vs 50.3 AU/mL, P < 0.001). In total, the sensitivity/specificity/PPV/NPV of *Aspergillus* IgG LFA was 65.1%/97.5%/95.4%/78.0%. The sensitivity and specificity of *Aspergillus* IgG LFA were equivalent to those of *Aspergillus* IgG ELISA with a 120 AU/mL cut-off, but exhibited significantly higher specificity (97.5% vs 87.5%, P = 0.021) compared to the ELISA with an 80 AU/mL cut-off. The consistency was strong among the two methods (P < 0.001, Kappa = 0.67/0.68). The sensitivities/specificities/PPVs/NPVs of *Aspergillus* IgG LFA were 57.6%/97.5%/90.5%/84.8% for patients with acute IA, and 73.3%/97.5%/91.7%/90.7% for patients with subacute IA, respectively. The “any-positive” strategy, which combined *Aspergillus* IgG LFA with sputum culture and serum galactomannan (GM), had a sensitivity/specificity/PPV/NPV of 81.1%/94.7%/95.6%/78.3%. The sensitivity/specificity/PPV/NPV of bronchoalveolar lavage fluid (BALF) GM was 65.0%/90.0%/92.9%/56.3%. When combined *Aspergillus* IgG LFA with BALF GM, the figures were 87.5%/85.0%/92.1%/77.3%.

**Conclusions:**

Compared to the *Aspergillus* IgG ELISA, the *Aspergillus* IgG LFA exhibits comparable or superior diagnostic efficiency in IA patients, while offering a faster and more convenient option for clinical diagnosis. The “any-positive” strategy of combined diagnosis with *Aspergillus* IgG LFA serves as a valuable supplement to current diagnostic approaches, particularly benefiting patients who cannot tolerate invasive bronchoscopic procedures.

## 1 Background

The latest epidemiological survey indicated that over 2.1 million people developed invasive aspergillosis (IA) annually, resulting in over 1.8 million of deaths each year (Denning, 2024). The diagnosis of IA primarily relies on host factor, clinical manifestation and mycological evidence (Ullmann et al., 2018; Donnelly et al., 2020; Bassetti et al., 2024). However, non-neutropenic patients with IA lack specificity in clinical symptoms and radiological manifestations, leaving challenges and rendering difficult diagnosis in clinical practice (Liu et al., 2021).

Non-neutropenic IA patients exhibit limited *Aspergillu*s vascular invasion, yielding low serum galactomannan (GM) antigen detection rates (Dagenais and Keller, 2009; Zhou et al., 2017). Conventional airway culture suffers from low sensitivity, prolonged processing (> 72 hours), and inability to discriminate infection from colonization (Baker and Denning, 2023). Although bronchoalveolar lavage fluid (BALF)-based diagnostics (GM, polymerase chain reaction [PCR]/next generation sequencing [NGS]) exhibit superior sensitivity, their invasiveness prevents their use in cardiopulmonary-compromised patients (Zhou et al., 2017; Baker and Denning, 2023; Zhu et al., 2023a). Therefore, it highlights the clinical need for non-invasive yet sensitive detection methods.

*Aspergillus* IgG antibody (*Aspergillus* IgG) is produced in response to *Aspergillus* infection (Delliere and Aimanianda, 2023). The detection of *Aspergillus* IgG in serum or plasma aids in diagnosing acute and subacute IA, but definitive diagnosis requires integration with clinical presentation and other corroborating evidence (Yu et al., 2020; Lu et al., 2023; Sheng et al., 2024). The available *Aspergillus* IgG assays include precipitin detection, counterimmunoelectrophoresis (CIE), enzyme-linked immunosorbent assay (ELISA) and immune chromatography technology (ICT), etc (Page et al., 2015; Baker and Denning, 2023). The *Aspergillus* IgG ELISA method has demonstrated its superiority in accuracy, stability and turn-around time (TAT) over the precipitin detection and CIE (Baxter et al., 2013; Page et al., 2015). Commercial *Aspergillus* IgG ELISA kits typically require a processing time of 2 to 4 hours (Page et al., 2015). Meanwhile, it is cost-effective to perform this test when an adequate number of samples are collected, which ultimately leads to a longer TAT. The detection of *Aspergillus* IgG using the ICT method has outstanding advantages, including the ability to detect a single sample, simple operation and a short TAT (less than 30 minutes) (Baker and Denning, 2023).

Timely identification and intervention of IA are vital for improving patient outcomes and reducing mortality (Page et al., 2015). However, current studies of *Aspergillus* IgG ICT primarily focus on patients with chronic pulmonary aspergillosis (CPA) (Piarroux et al., 2019; Stucky Hunter et al., 2019; Rozaliyani et al., 2020; Ray et al., 2022; Singh et al., 2022; Zhu, 2023b). In this study, we evaluated the performance of a novel *Aspergillus* IgG lateral flow assay (LFA) based on fluorescence ICT for the diagnosis of acute and subacute IA in non-neutropenic patients.

## 2 Methods

### 2.1 Patients and samples

This retrospective, controlled study included a total of 143 non-neutropenic patients (consisting of 63 cases with IA and 80 controls), identified at Nanjing Drum Tower Hospital and Nanjing Jinling Hospital from June 2018 to September 2023. Out of the 63 patients with IA, 33 (52.4%) had acute IA and 30 (47.6%) had subacute IA. There were 3 (9.1%) patients with proven diagnosis and 30 (90.9%) patients with probable diagnosis in the acute IA group. There were 1 (3.3%) patient with proven diagnosis and 29 (96.7%) patients with probable diagnosis in the subacute IA group. The control group consisted of 50 patients with proved community acquired pneumonia (CAP) and 30 healthy volunteers without any evidence of *Aspergillus* infection or colonization. All diagnoses were based on clinical evidence and verified by experienced specialists. Blood samples used in this study were obtained prior to the initiation of antifungal therapy. These samples were the surplus plasma from previous clinical studies and clinical routine tests, which were stored in a −80°C refrigerator.

### 2.2 The diagnostic criteria of IA

The diagnosis of IA was established according to the 2020 guideline from EORTC/MSG (Donnelly et al., 2020), with minor modifications. Proven IA required histopathologic evidence for *Aspergillus* hyphae in sterile lung specimens. Probable IA needed evidence of at least one host factor, clinical manifestation and mycological evidence. Host factors included but were not limited to solid organ transplant, prolonged use of corticosteroids or immunosuppressive agents, acute respiratory distress syndrome (ARDS), chronic obstructive pulmonary disease (COPD), severe influenza, diabetes and/or malnutrition (Ullmann et al., 2018). The radiological features consisted of dense, well-circumscribed lesions with or without a halo sign, an air crescent sign, cavity and/or consolidation. Mycological evidence included positive results of GM test, PCR test and/or sputum/BALF/bronchial brush/aspirate culture (Donnelly et al., 2020). The diagnosis of non-neutropenic IA for intensive care unit (ICU) patients relied on the 2024 consensus definitions from the study group of the ESGCIP, EFISG, ESICM, ECMM, MSGERC, ISAC and ISHAM (Bassetti et al., 2024). Host factors included COVID-19, solid tumors, moderate/severe COPD, influenza, decompensated liver cirrhosis and/or human immunodeficiency virus (HIV). The radiologic findings included pulmonary infiltrate and/or cavity. Mycological evidence included positive results of GM test and/or BALF culture. The detection of *Aspergillus* nucleic acid in this study was conducted using NGS (Zhu et al., 2023a). *Aspergillus* IgG was not included as one of the diagnostic criteria for IA.

### 2.3 *Aspergillus* IgG antibody assay

**Figure 1:**
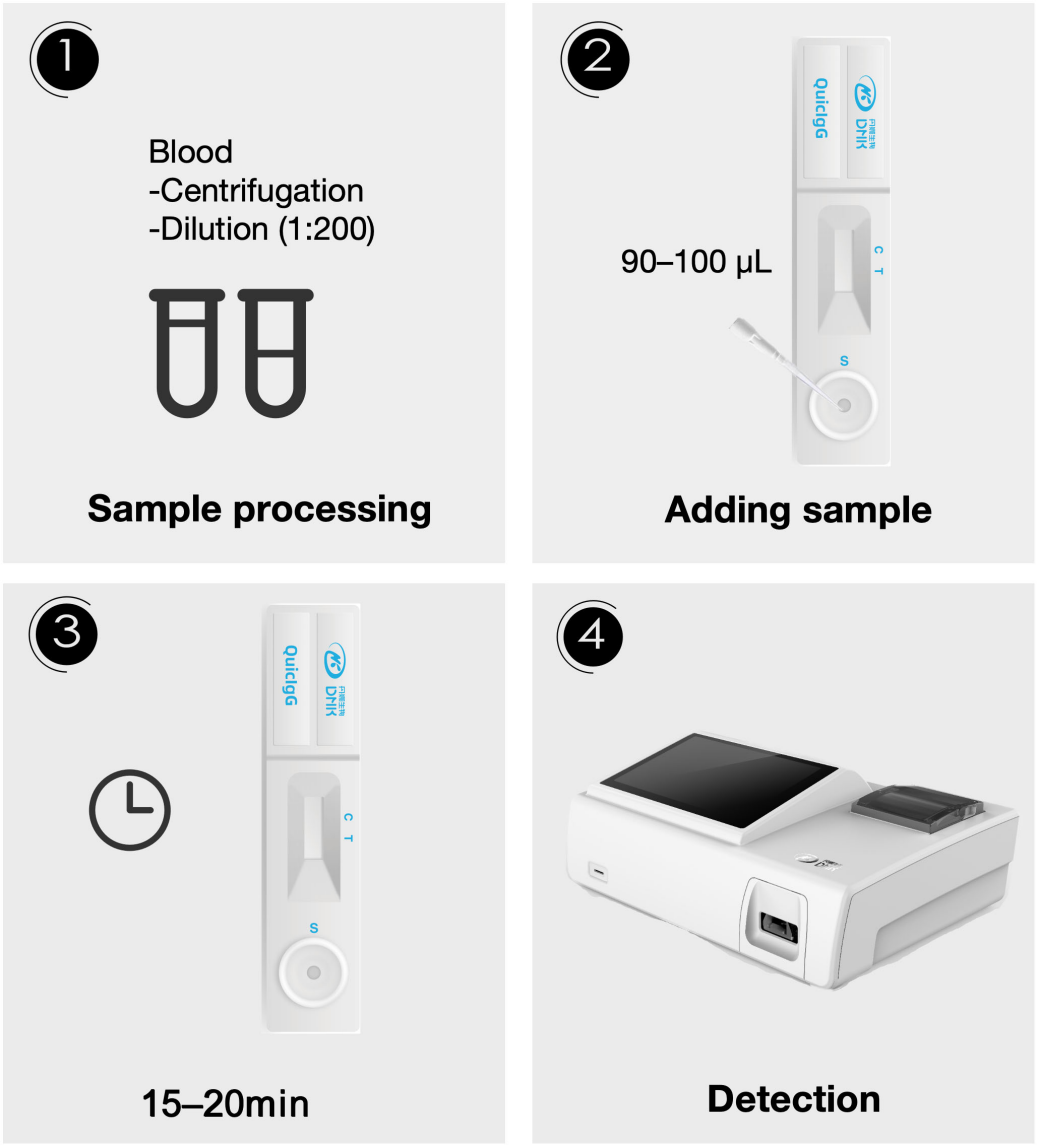
The flow chart of the *Aspergillus* IgG assay via fluorescence lateral flow technique. Step 1: Blood samples were centrifuged, then the plasma was diluted 1:200. Step 2: 90–100 μL of the diluted sample was added to the sample well of the test card. Step 3: A 15–20 minute incubation period was allowed. Step 4: Detection was performed using an optical device.

The *Aspergillus* fluorescence IgG LFA (Dynamiker, Tianjin, China) was utilized for the semi-quantitative detection of *Aspergillus* galactomannan IgG antibody in human plasma samples. The test kit was stored in a refrigerator at 4°C. Prior to use, the required number of test cards, reagents and plasma samples were brought to room temperature. Plasma samples were diluted to 1:200 using phosphate buffered saline (PBS) containing protein. Subsequently, 90–100 μL of the diluted sample were added to the sample well of each test card. Within 15 to 20 minutes, fluorescence signal was obtained by scanning the detection area with specialized fluorescence immunoanalyzer (Dynamiker, Tianjin, China). Then, the result could be read in digital form (**Figure 1**). According to the manufacturer’s instructions, results greater than 135 AU/mL were considered positive, results less than 135 AU/mL were considered negative. The minimum detection limit of this reagent was 10 AU/mL.

The *Aspergillus* fumigatus IgG ELISA test kit from Dynamiker (Tianjin, China) was utilized for the quantitative detection of *Aspergillus* IgG in plasma samples. Plasma samples were diluted to 1:1000 and tested following the manufacturer’s instructions. Results greater than 120 AU/mL were considered positive, results less than 80 AU/mL were considered negative, and results between 80 and 120 AU/mL were deemed borderline. The minimum detection limit of this reagent was 31.3 AU/mL.

### 2.4 GM test and culture

Detection of GM in BALF and serum was conducted using an enzyme-linked immunosorbent double antibody sandwich assay (Bio-Rad Laboratories, CA, USA) by the clinical central laboratories. Qualified expectorated sputum and BALF samples were cultured on Sabouraud dextrose agar by the clinical microbiology laboratories.

### 2.5 Statistical analysis

SPSS 27.0 (SPSS Inc., Chicago, IL, USA) and R statistical software 4.2.3 (R Foundation for Statistical Computing, Vienna, Austria) were used for data analysis. GraphPad Prism 10 (GraphPad Software, CA, USA) was used to draw graphs. Categorical variables were analyzed using Pearson’s chi-squared test or Fisher’s exact test, with results presented as frequency counts (percentages). McNemar’s test was used for paired categorical variables. Continuous data were analyzed using the Mann-Whitney U test for comparisons between two groups, and the Kruskal-Walis test for comparisons involving more than two groups. Results were presented as the median with the interquartile range (IQR). Receiver operating characteristic (ROC) curve analysis was performed to obtain sensitivity, specificity, Youden index (sensitivity+specificity-1) and optimal threshold. The association between *Aspergillus* IgG ELISA and *Aspergillus* IgG LFA was assessed by Spearman’s rank correlation coefficient and Kappa consistency test (0–0.2, very weak; 0.21–0.4, weak; 0.41–0.6, moderate; 0.61–0.8, strong; and 0.81–1, almost perfect). *P* value < 0.05 was considered to be statistically significant.

## 3 Results

### 3.1 The characteristics of patients with IA

The characteristics of the study population are shown in **Table 1**. Patients with IA had a median age of 64 (IQR 56-72) years, and 76.2% of them were male. The positive rate of *Aspergillus* IgG LFA (65.1%) was significantly higher than that of serum GM (27.1%, P < 0.001) and sputum culture (30.4%, P = 0.001), comparable to BALF GM (65.0%, P = 0.824) and *Aspergillus* IgG ELISA (69.8%, P = 0.549), and significantly lower than that of BALF NGS (88.9%, P = 0.035). The positive rates of only *A. fumigatus* infection and other *Aspergillus* infection except *A. fumigatus* were 67.9% and 60.0%, respectively (P = 0.709)(**Table 2**).

**Table 1:** The characteristics of invasive aspergillosis group, community acquired pneumonia group and healthy control.

Characteristics	IA (N=63)	CAP (N=50)	Healthy control (N=30)	*P* value
Gender, n (%)				
Male	48 (76.2)	33 (66.0)	20 (66.7)	0.431
Female	15 (23.8)	17 (34.0)	10 (33.3)	
Age, median (IQR)	64 (56-72)	65.5 (55.8-72)	63 (58-75)	0.830
ICU admission, n (%)	29 (46.0)	15 (30.0)		0.083
Underlying diseases, n (%)				
Chronic obstructive pulmonary disease	8 (12.7)	13 (26.0)		0.071
Bronchiectasis	9 (14.3)	6 (12.0)		0.772
Lung cancer	12 (19.0)	6 (12.0)		0.309
Other solid organ tumor	6 (9.5)	7 (14.0)		0.459
Hematologic malignancy	3 (4.8)	0 (0)		0.330
COVID-19	4 (6.3)	0 (0)		0.193
Diabetes mellitus	15 (23.8)	7 (14.0)		0.191
Organ transplantation	3 (4.8)	1 (2.0)		0.782
Use of corticosteroids	23 (36.5)	8 (16.0)		0.015
Use of immunosuppressants	9 (14.3)	2 (4.0)		0.130
CT thorax findings, n (%)				
Inflitrate/s	52 (82.3)	39 (78.0)		0.545
Consolidation	19 (30.2)	11 (22.0)		0.329
Cavity	19 (30.2)	2 (4.0)		0.000
Nodule	28 (44.4)	31 (62.0)		0.064
Air crescent sign	1 (1.6)	0 (0)		1.000
Pleural thickening	25 (39.7)	16 (32.0)		0.399
Pleural effusion	32 (50.8)	21 (42.0)		0.352
Blood indexs, median (IQR)				
White blood cell count (10^9^cells/L)	8.2 (5.2-12.2)	7.1 (5.0-9.4)		0.128
Neutrophil count (10^9^cells/L)	6.5 (3.6-10.6)	5.3 (3.4-7.5)		0.187
Lymphocyte count (10^9^cells/L)	0.9 (0.7-1.5)	1.1 (0.7-1.4)		0.714
C-reactive protein (mg/L)	65.4 (8.9-156.9)	15.9 (2.5-56.0)		0.004
Pocalcitonin (ng/mL)	0.2 (0.1-0.5)	0.1 (0.0-0.3)		0.003
Mycological findings, n (%)				
Positive BALF NGS	32/36 (88.9)	0/8 (0)		
*Aspergillus* IgG ELISA ≥ 80 AU/mL	44 (69.8)	9 (18.0)	1 (3.3)	
*Aspergillus* IgG LFA ≥ 135 AU/mL	41 (65.1)	2 (4.0)	0 (0)	
BALF GM ≥ 1.0 ODI	26/40 (65.0)	2/20 (10.0)		
Positive blood NGS	7/11 (63.6)	0/2 (0)		
*Aspergillus* IgG ELISA ≥ 120 AU/mL	38 (60.3)	0 (0)	0 (0)	
Serum GM ≥ 0.5 ODI	30/59 (50.8)	3/45 (6.7)		
Positive BALF culture	12/36 (33.3)	0/16 (0)		
Positive sputum culture	17/56 (30.4)	0/42 (0)		
Serum GM ≥ 1.0 ODI	16/59 (27.1)	1/45 (2.2)		

IA, invasive aspergillosis; CAP, community acquired pneumonia; IQR, interquartile range; ICU, intensive care unit; CT, computed tomography; BALF, bronchoalveolar lavage fluid; NGS, next generation sequencing; ELISA, enzyme-linked immunosorbent assay; LFA, lateral flow assay; GM, galactomannan; ODI, optical density index.

**Table 2:** The positive rates of *Aspergillus* IgG lateral flow assay in invasive aspergillosis patients with different types of *Aspergillus* infections.

Types of *Aspergillus* infections	N	LFA +, n ≥ 135 AU/mL	Positive rates,%
Only *A. fumigatus*	28	19	67.9
*A. fumigatus*+*A. flavus*	2	2	
*A. fumigatus*+*A*. *niger*	1	0	
*A. fumigatus*+*A. flavus*+*A. oryzae*	1	1	
Only *A. flavus*	7	4	57.1
Only *A. niger*	1	1	
Only *A. terreus*	1	0	
*A. flavus*+*A. oryzae*	1	1	
Except for *A. fumigatus*	10	6	60.0

LFA, lateral flow assay; *A*, *Aspergillus.*

### 3.2 The correlation between *Aspergillus* IgG LFA and *Aspergillus* IgG ELISA

**Figure 2:**
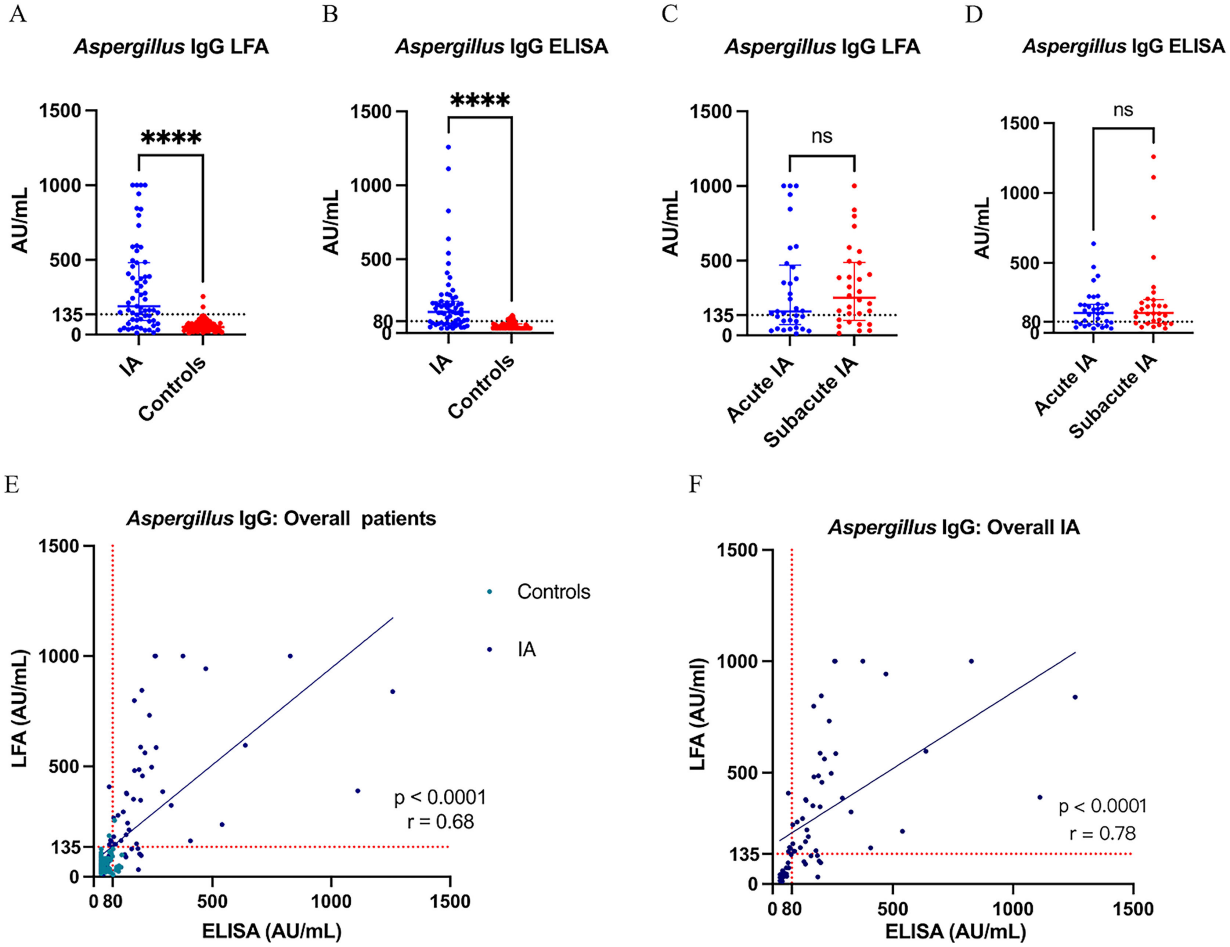
**(A, B)** The levels of *Aspergillus* IgG LFA and *Aspergillus* IgG ELISA in overall IA and control groups; **(C, D)** The levels of *Aspergillus* IgG LFA and *Aspergillus* IgG ELISA in acute IA and subacute IA groups; **(E, F)** Scatter diagram of *Aspergillus* IgG LFA vs *Aspergillus* IgG ELISA in overall and IA patients. **** P < 0.001; ns, not significant (P > 0.05); the value of r represented correlation coefficient. LFA, lateral flow assay; IA, invasive aspergillosis; ELISA, enzyme-linked immunosorbent assay.

The levels of *Aspergillus* IgG LFA and *Aspergillus* IgG ELISA in patients with IA were significantly higher than those in control groups, respectively (Median[IQR]: 190.5 [94.5-481.4] vs 50.3 [31.4-75.2] AU/mL for LFA, P <0.001; 143.0 [67.3-215.7] vs 36.8 [31.3-60.8] AU/mL for ELISA, P< 0.001) (**Figures 2A, B**). There were no significant differences in the levels of *Aspergillus* IgG LFA (160.5[72.5-469.2] vs 251.7[100.0-488.4] AU/mL, P = 0.466) and *Aspergillus* IgG ELISA (143.0[61.7-204.1] vs 144.1[69.8-237.4] AU/mL, P = 0.527) between the acute IA group and the subacute IA group (**Figures 2C, D**).

For all 143 patients of this study, the *Aspergillus* IgG LFA showed a strong correlation with the *Aspergillus* IgG ELISA (P < 0.0001, r = 0.68) (**Figure 2E**). The consistency was strong among the two methods (P < 0.001, overall agreement = 85.3%, Kappa = 0.68, cut-off value of *Aspergillus* IgG = 80AU/mL; P < 0.001, overall agreement = 86.7%, Kappa = 0.67, cut-off value of *Aspergillus* IgG = 120AU/mL) (**Table 3**).

**Table 3:** Consistency analysis of *Aspergillus* IgG enzyme-linked immunosorbent assay and *Aspergillus* IgG lateral flow assay in all patients..

Variables		*Aspergillus* IgG ELISA			Total
		Positive ≥ 120 AU/mL	Weak positive 80-120 AU/mL	Negative <80 AU/mL	
*Aspergillus* IgG LFA	Positive ≥ 135 AU/mL	31(21.7)	7(4.9)	5(3.5)	43
	Negative < 135 AU/mL	7(4.9)	9(6.3)	84(58.7)	100
Total		38	16	89	143

ELISA, enzyme-linked immunosorbent assay; LFA, lateral flow assay.

For the IA group, the *Aspergillus* IgG LFA showed a strong correlation with the *Aspergillus* IgG ELISA (P < 0.0001, r = 0.78) (**Figure 2F**). The consistency was moderate among the two methods (P < 0.001, overall agreement = 82.5%, Kappa = 0.60, cut-off value of *Aspergillus* IgG = 80AU/mL; P = 0.001, overall agreement = 73.0%, Kappa = 0.43, cut-off value of *Aspergillus* IgG = 120AU/mL) (**Table 4**).

**Table 4:** Consistency analysis of *Aspergillus* IgG enzyme-linked immunosorbent assay and *Aspergillus* IgG lateral flow assay in invasive aspergillosis patients.

Variables		*Aspergillus* IgG ELISA			Total
		Positive ≥ 120 AU/mL	Weak positive 80-120 AU/mL	Negative <80 AU/mL	
*Aspergillus* IgG LFA	Positive ≥ 135 AU/mL	31(49.2)	6(9.5)	4(6.3)	41
	Negative < 135 AU/mL	7(11.1)	0(0)	15(23.8)	22
Total		38	6	19	63

ELISA, enzyme-linked immunosorbent assay; LFA, lateral flow assay.

### 3.3 Comparing the diagnostic performance of *Aspergillus* IgG LFA with *Aspergillus* IgG ELISA

**Figure 3:**
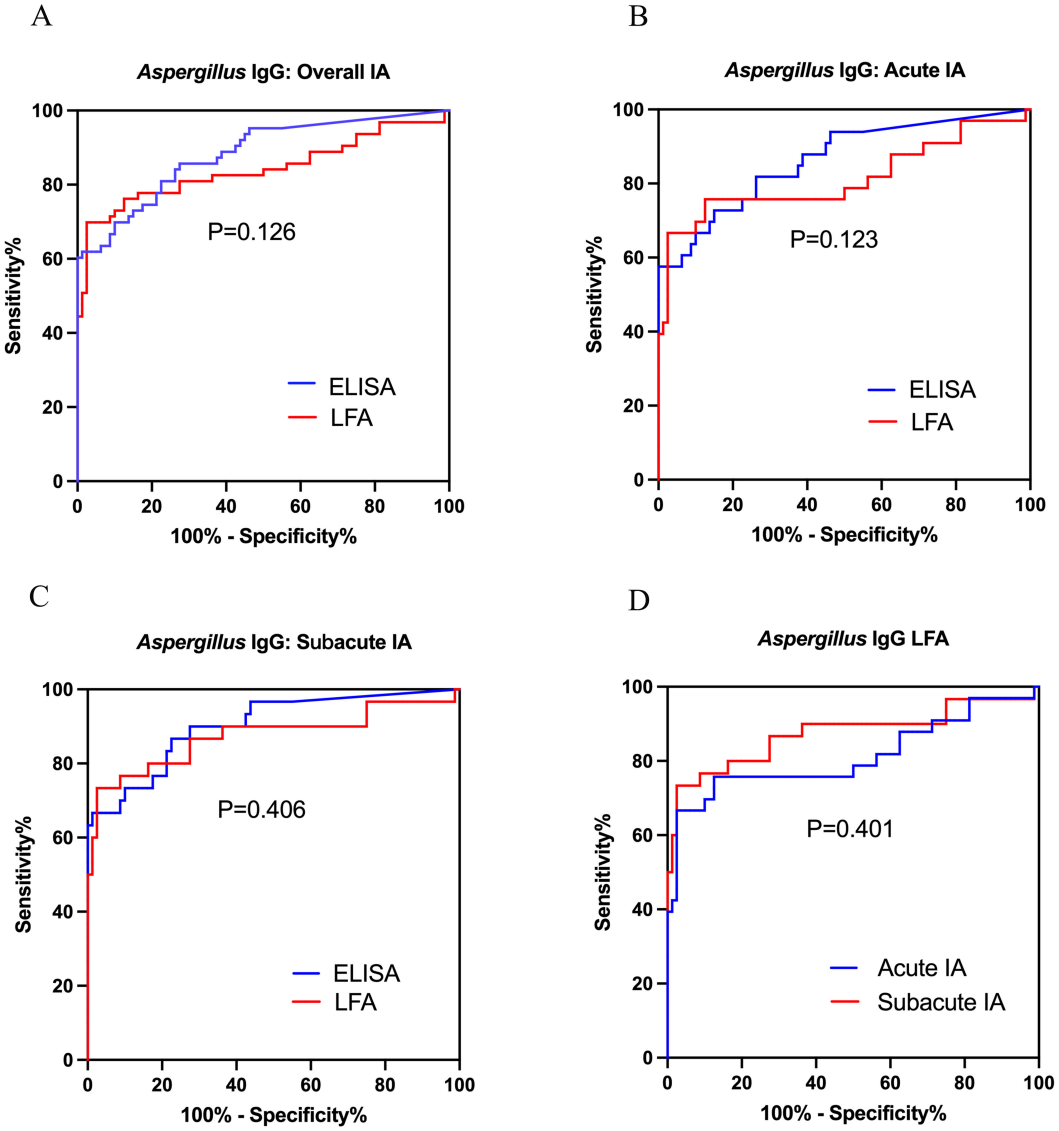
Receiver operating characteristic curves were used to evaluate the performance of *Aspergillus* IgG LFA and ELISA in diagnosing acute IA, subacute IA or overall IA group, compared to CAP group and healthy control. **(A)** LFA vs ELISA in overall IA; **(B)** LFA vs ELISA in acute IA; **(C)** LFA vs ELISA in subacute IA; **(D)** LFA in acute IA and subacute IA. IA, invasive aspergillosis; ELISA, enzyme-linked immunosorbent assay; LFA, lateral flow assay.

The area under ROC curves of *Aspergillus* IgG LFA vs ELISA were 0.842 (95% CI: 0.768, 0.915) vs 0.882 (95% CI: 0.825, 0.938) for overall IA patients (**Figure 3A**), 0.812 (95% CI: 0.705, 0.919) vs 0.863 (95% CI: 0.783, 0.944) for patients with acute IA (**Figure 3B**), and 0.874 (95% CI: 0.780, 0.967) vs 0.903 (95% CI: 0.834, 0.971) for patients with subacute IA (**Figure 3C**), respectively. There were no significant differences between the two methods across the three groups (P were 0.126, 0.123 and 0.406, respectively).

In total, the sensitivity and specificity of *Aspergillus* IgG LFA were equivalent to these of the *Aspergillus* IgG ELISA with a cut-off value of 120 AU/mL (65.1% vs 60.3% for sensitivity, P = 0.629; 97.5% vs 100% for specificity, P = 0.497). Compared to the *Aspergillus* IgG ELISA with a cut-off value of 80 AU/mL, the *Aspergillus* IgG LFA had equivalent sensitivity (65.1% vs 69.8% for sensitivity, P = 0.549), but significantly higher specificity (97.5% vs 87.5% for specificity, P = 0.021)(**Table 5**).

**Table 5:** The diagnostic performances of *Aspergillus* IgG lateral flow assay and *Aspergillus* IgG enzyme-linked immunosorbent assay.

Variables	LFA ≥ 135 AU/mL	ELISA ≥ 80 AU/mL	ELISA ≥ 120 AU/mL
Overall IA (n=63)			
Sensitivity,% (95% CI)	65.1(57.3,72.9)	69.8(62.3,77.4)	60.3(52.3,68.3)
Specificity,% (95% CI)	97.5(94.9,100.1)	87.5(82.1,92.9)	100(100,100)
PPV,% (95% CI)	95.4(91.9,98.8)	81.5(75.1,87.9)	100(100,100)
NPV,% (95% CI)	78.0(71.2,84.8)	78.7(71.9,85.4)	76.2(69.2,83.2)
Youden's index (95% CI)	0.626(0.547,0.705)	0.573(0.492,0.655)	0.603(0.523,0.683)
Accuracy,% (95% CI)	83.2(77.1,89.3)	79.7(73.1,86.3)	82.5(76.3,88.7)
Acute IA (n=33)			
Sensitivity,% (95% CI)	57.6(48.5,66.7)	66.7(58.0,75.4)	57.6(48.5,66.7)
Specificity,% (95% CI)	97.5(94.6,100.4)	87.5(81.4,93.6)	100(100,100)
PPV,% (95% CI)	90.5(85.1,95.9)	68.8(60.2,77.3)	100(100,100)
NPV,% (95% CI)	84.8(78.2,91.4)	86.4(80.1,92.7)	85.1(78.5,91.7)
Youden's index (95% CI)	0.551(0.459,0.643)	0.542(0.450,0.634)	0.576(0.485,0.667)
Accuracy,% (95% CI)	85.8(79.4,92.3)	81.4(74.2,88.6)	87.6(81.5,93.7)
Subacute IA (n=30)			
Sensitivity,% (95% CI)	73.3(65.1,81.6)	73.3(65.1,81.6)	63.3(54.3,72.3)
Specificity,% (95% CI)	97.5(94.6,100.4)	87.5(81.3,93.7)	100(100,100)
PPV,% (95% CI)	91.7(86.5,96.8)	68.8(60.1,77.4)	100(100,100)
NPV,% (95% CI)	90.7(85.3,96.1)	89.7(84.1,95.4)	87.9(81.8,94.0)
Youden's index (95% CI)	0.708(0.623,0.793)	0.608(0.517,0.700)	0.633(0.543,0.723)
Accuracy,% (95% CI)	90.9(85.5,96.3)	83.6(76.7,90.6)	90.0(84.4,95.6)

LFA, lateral flow assay; ELISA, enzyme-linked immunosorbent assay; IA, invasive aspergillosis; CI, confidence interval; PPV, positive predictive value; NPV, negative predictive value.

### 3.4 Comparing the diagnostic performance of *Aspergillus* IgG LFA between patients with acute IA and subacute IA

The ROC curves of *Aspergillus* IgG LFA had no significant differences between patients with acute and subacute IA (AUC0.812 [95% CI: 0.705, 0.919] vs 0.874 [95% CI: 0.780, 0.967], P =0.401) (**Figure 3D**). The sensitivities, PPVs and NPVs of the *Aspergillus* IgG LFA were equivalent between patients with acute and subacute IA (57.6% vs 73.3% for sensitivity, P = 0.190; 90.5% vs 91.7% for PPV, P = 1.000; 84.8% vs 90.7% for NPV, P =0.231) (**Table 5**).

### 3.5 The optimal cut-off values of *Aspergillus* IgG LFA for patients with acute IA, subacute IA and overall IA

According to the ROC curves, the optimal cut-off values were determined to be 126, 136 and 126 AU/mL for patients with acute IA, subacute IA and overall IA, respectively (**Figure 3**). The corresponding sensitivities/specificities/PPVs/NPVs were as follows: 66.7%/97.5%/91.7%/87.6% for patients with acute IA, 73.3%/97.5%/91.7%/90.7% for patients with subacute IA, and 69.8%/97.5%/95.7%/80.4% for overall IA patients, respectively.

### 3.6 The evaluation of the combined diagnostic performance of *Aspergillus* IgG LFA in patients with IA

A total of 53 IA patients and 38 CAP patients had *Aspergillus* IgG LFA, sputum culture, and serum GM results. For these patients, the use of an “any-positive” strategy, which combined sputum culture with serum GM, had a sensitivity of 43.4% (23/53) and a specificity of 97.4% (37/38). When combined with *Aspergillus* IgG LFA, the sensitivity significantly improved to 81.1% (43/53, P < 0.001), while maintaining a comparable specificity of 94.7% (36/38, P = 1.000).

A total of 40 IA patients and 20 CAP patients had *Aspergillus* IgG LFA and BALF GM results. For these patients, the sensitivity and specificity of BALF GM were 65.0% (26/40) and 90.0% (18/20), respectively. The use of an “any-positive” strategy, which combined *Aspergillus* IgG LFA with BALF GM, significantly improved sensitivity to 87.5% (35/40, P = 0.004), while maintaining comparable specificity to that of BALF GM alone at 85.0% (17/20, P = 1.000).

## 4 Discussion

This study demonstrated that Dynamiker *Aspergillus* IgG LFA had good diagnostic performance in non-neutropenic patients with acute and subacute IA (**Figure 3**), showing equivalent sensitivity and specificity to the *Aspergillus* IgG ELISA with a 120 AU/mL cut-off, but exhibiting significantly higher specificity compared to the ELISA with an 80 AU/mL cut-off (**Table 5**). This method demonstrated comparable or superior efficiency to the *Aspergillus* IgG ELISA, offering a novel and effective tool for the detection of *Aspergillus* IgG. The combined diagnosis with *Aspergillus* IgG LFA serves as a valuable supplement to current diagnostic approaches (**Table 6**).

**Table 6:** Combined diagnostic performances of *Aspergillus* IgG lateral flow assay with other non-invasive tests (sputum culture, serum galactomannan) or bronchoalveolar lavage fluid galactomannan in invasive aspergillosis patients.

Variables	Sputum culture/serum GM	LFA/sputum culture/serum GM	LFA and sputum culture/serum GM	BALF GM	LFA/BALF GM	LFA and BALF GM
Sensitivity, % (95% CI)	43.4 (33.2,53.6)	81.1 (73.1,89.2)	26.4 (17.4,35.8)	65.0 (52.9,77.1)	87.5 (79.1,95.9)	37.5 (25.3,49.8)
Specificity, % (95% CI)	97.4 (94.1,100.7)	94.7 (90.2,99.3)	100 (100,100)	90.0 (82.4,97.6)	85.0 (76.0,94.0)	100 (100,100)
PPV, % (95% CI)	95.8 (91.7,99.9)	95.6 (91.3,99.8)	100 (100,100)	92.9 (86.3,99.4)	92.1 (85.3,98.9)	100 (100,100)
NPV, % (95% CI)	55.2 (45.0,65.4)	78.3 (69.8,86.7)	49.4 (39.1,59.6)	56.3 (43.7,68.8)	77.3 (66.7,87.9)	44.4 (31.9,57.0)
Youden’s index (95% CI)	0.408 (0.307,0.509)	0.759 (0.671,0.847)	0.264 (0.174,0.355)	0.550 (0.424,0.676)	0.725 (0.612,0.838)	0.375 (0.253,0.498)
Accuracy, % (95% CI)	65.9 (56.2,75.7)	86.8 (79.9,93.8)	57.1 (47.0,67.3)	73.3 (62.1,84.5)	86.7 (78.1,95.3)	58.3 (45.9,70.8)

The cut-off value of *Aspergillus* IgG LFA was 135 AU/mL; The cut-off value of serum GM and BALF GM was 1.0 ODI. LFA, lateral flow assay; GM, galactomannan; BALF, bronchoalveolar lavage fluid; CI, confidence interval;PPV, positive predictive value; NPV, negative predictive value; ODI, optical density index.

The diagnosis of IA in non-neutropenic patients typically depends heavily on positive results of BALF GM, in which sensitivity and specificity were 55%-74% and 90%-99%, respectively (Zhou et al., 2017; Dai et al., 2021; Lu et al., 2023). In this study, the sensitivity and specificity of the BALF GM were 65.0% and 90.0%, respectively (**Table 6**). Additionally, the efficacy of BALF PCR in diagnosing IA is supported by sufficient evidence, but its main focus is on the population of hematological malignancies (HM) and/or hematopoietic stem cell/solid organ transplantation (HSCT/SOT). A meta-analysis showed that PCR had higher sensitivity for the diagnosis of IA in non-neutropenic patients such as COPD, solid tumors and autoimmune diseases with prolonged corticosteroid therapy, compared to those with HM and/or HSCT/SOT (88% vs 68%, P < 0.001) (Han et al., 2023). In this study, the sensitivity of BALF NGS in non-neutropenic IA patients was 88.9% (**Table 1**). The *Aspergillu*s IgG LFA exhibited a sensitivity of 65.1%, which, although lower than that of BALF NGS, was comparable to BALF GM testing. As a non-invasive method, it offers advantages in procedural safety, patient tolerance, and clinical workflow simplification.

Among noninvasive tests, plasma *Aspergillus* IgG ELISA (Lu et al., 2023) and LFA (**Table 1**) had higher sensitivities than serum GM and sputum culture in non-neutropenic patients with IA, respectively. The sensitivity and specificity of *Aspergillus* IgG ELISA were 50%-69% and 77%-89% in non-neutropenic patients with IA, respectively (Yu et al., 2020; Lu et al., 2023; Sheng et al., 2024). In this study, the sensitivity and specificity of *Aspergillus* IgG ELISA for non-neutropenic patients with acute and subacute IA were 60%-69% and 87%-100%, respectively (**Table 3**). Previous studies included patients with suspected IA, rather than randomly selected patients with CAP, so the specificity was lower than in this study. Piarroux et al. found that the sensitivity and specificity of the LDBio *Aspergillus* ICT (Lyons, France) in patients with acute and subacute IA were 67% (14/21) and 96%, respectively (Piarroux et al., 2019). In our study, we observed the sensitivity and specificity of the Dynamiker *Aspergillus* IgG LFA in non-neutropenic patients with acute and subacute IA were 65.1% (41/63) and 97%, respectively (**Table 5**).

The diagnostic role of *Aspergillus* IgG LFA in non-neutropenic patients with IA is rarely reported. In this study, we demonstrated that *Aspergillus* IgG LFA had reliable diagnostic value for non-neutropenic patients with acute and subacute IA (**Figure 3**). According to the manufacturer’s instructions, 135 AU/mL was determined as the cut-off value. In non-neutropenic patients with IA, it was in better agreement with the ELISA threshold of 80 AU/mL (**Table 4**). In this study, the optimal cut-off values for non-neutropenic patients with IA were determined to be 126 AU/mL (**Figure 3**).

The LDBio *Aspergillus* IgG ICT and Era Biology *Aspergillus* IgG LFA (Tianjin, China) serve as tools for the qualitative detection of *Aspergillus* IgG, and the results are determined visually (Piarroux et al., 2019; Stucky Hunter et al., 2019; Rozaliyani et al., 2020; Ray et al., 2022; Singh et al., 2022; Zhu, 2023b). Stucky et al. reported that the sensitivities of the LDBio *Aspergillus* IgG ICT and ELISA method were 91.6% and 80.5%, respectively; however, the accuracy of quantifying *Aspergillus* IgG based on the test line strength of ICT was unreliable (Stucky Hunter et al., 2019). Ray et al. determined that the sensitivities of the *Aspergillus* IgG ELISA and LDBio *Aspergillus* IgG ICT were 82.4% and 67.6%, respectively, with specificities of 82% and 81% (Ray et al., 2022). Zhu et al. reported that the *Aspergillus* IgG ELISA and Era Biology *Aspergillus* IgG LFA had sensitivities of 55.2% and 54.3%, and specificities of 89.1% and 92.7%, respectively (Zhu, 2023b). Therefore, the diagnostic performance of *Aspergillus* IgG ELISA and LFA is still controversial. At present, relevant studies have taken CPA patients as research objects. There are no studies comparing the diagnostic effectiveness of the two methods in non-neutropenic patients with IA.

Alternatively, the Dynamiker *Aspergillus* IgG LFA provides a feasible option for the semi-quantitative detection of *Aspergillus* IgG, and the results can be read in digital form. The *Aspergillus* IgG LFA had a strong correlation with the *Aspergillus* IgG ELISA (**Figures 2E, F**). The sensitivity and specificity of the Dynamiker *Aspergillus* IgG LFA were comparable to those of the *Aspergillus* IgG ELISA when using a cut-off value of 120 AU/mL. However, the Dynamiker *Aspergillus* IgG LFA exhibited significantly higher specificity when the cut-off value of *Aspergillus* IgG ELISA was set at 80 AU/mL (**Table 5**).

While the detection of *Aspergillus* IgG can be a valuable component in diagnosing IA, due to issues such as specificity problems and insufficient sensitivity in acute infections (**Table 5**), it is generally not sufficient as a standalone test for a definitive diagnosis, particularly in non-neutropenic patients. Clinical interpretation requires the integration of various diagnostic modalities, including radiographic findings, other microbiological evidence, and host risk factor assessment. Therefore, we evaluated the diagnostic value of combining *Aspergillus* IgG LFA with non-invasive examinations and BALF GM testing (**Table 6**). Employing the “any-positive” strategy demonstrated that this approach significantly improved the sensitivity of existing diagnostic methods while maintaining comparable specificity. This combined strategy serves as a valuable supplement to current diagnostic approaches, particularly benefiting cardiopulmonary-compromised patients who cannot tolerate invasive bronchoscopic procedures.

Currently, *Aspergillus* GM LFA is the most frequently reported LFA reagent for the detection of IA. Numerous studies (Mercier et al., 2020; Jani et al., 2021; Mercier et al., 2021) have demonstrated its superior performance in detecting BALF specimens from patients with HM and/or allogeneic stem cell transplantation. In IA patients without HM, the sensitivity and specificity of serum *Aspergillu*s GM LFA were reported to be 50% and 93%, respectively (Alhan et al., 2023); the sensitivity and specificity of BALF *Aspergillus* GM LFA were 58%-69% and 68%-75%, respectively (Jenks et al., 2019). Consequently, the diagnostic performance of plasma *Aspergillu*s IgG LFA in non-neutropenic IA seems comparable to that of BALF *Aspergillus* GM LFA. However, further research is required for a comprehensive comparison.

The limitations of this study are attributed to the retrospective study design, samll sample size, and the limited availability of clinical data. A larger prospective multicenter study is necessary to further validate the performance of *Aspergillus* IgG LFA in broader populations. This study defines diagnostic cut-off values of *Aspergillus* IgG LFA for patients with IA that need to be further validated in a new IA cohort. Of course, *Aspergillus* IgG LFA also has potential limitations, such as cross-reactivity with other fungal infections, inability to detect low concentrations of IgG antibodies, and differences among different manufacturers, etc. In addition, there are few studies comparing the diagnostic efficiency of various *Aspergillus* IgG ICT/LFA detection kits, so comparison of the diagnostic performance of existing kits is a necessary aspect for future work.

## 5 Conclusion

In summary, the Dynamiker *Aspergillus* IgG LFA represents a rapid, user-friendly, and highly effective auxiliary tool for the clinical diagnosis of non-neutropenic patients with IA.

## Data Availability

The raw data supporting the conclusions of this article will be made available by the authors, without undue reservation.
