# Correction: Evaluation of a novel *Aspergillus* IgG lateral flow assay for the diagnosis of non-neutropenic patients with acute and subacute invasive aspergillosis

**DOI:** 10.3389/fcimb.2025.1659574

**Published:** 2025-07-25

**Authors:** Yajie Lu, Huanhuan Zhong, Yujie Wang, Chao Sun, Yuanyuan Li, Yuchen Cai, Xiaomin Cai, Jiamei Wang, Jinjin Zhong, Xin Su

**Affiliations:** ^1^ Department of Respiratory and Critical Care Medicine, Nanjing Drum Tower Hospital, Affiliated Hospital of Medical School, Nanjing University, Nanjing, China; ^2^ Department of Respiratory and Critical Care Medicine, The Second Affiliated Hospital of Soochow University, Suzhou, China; ^3^ Department of Respiratory and Critical Care Medicine, Nanjing Jinling Hospital, Affiliated Hospital of Medical School, Nanjing University, Nanjing, China; ^4^ Department of Respiratory and Critical Care Medicine, Nanjing Jinling Hospital, Medical School, Nanjing Medical University, Nanjing, China

**Keywords:** acute invasive aspergillosis, subacute invasive aspergillosis, non-neutropenic patients, *Aspergillus* IgG lateral flow assay, diagnosis

The title of this article was erroneously given as: “Evaluation of a novel aspergillus IgG lateral flow assay for the diagnosis of non-neutropenic patients with acute and subacute invasive aspergillosis”. The correct title of the article is “Evaluation of a novel *Aspergillus* IgG lateral flow assay for the diagnosis of non-neutropenic patients with acute and subacute invasive aspergillosis”.

“Zhu, R. Z., Cheng, J., Luo, Y., Qiu, W., Huang, J., Jiang, Y., et al. (2023b). Diagnostic

laboratory features and performance of an aspergillus IgG lateral flow assay in a chronic

pulmonary aspergillosis cohort. Microbiol. Spectr. 11, e0026423. doi: 10.1128/

spectrum.00264-23]”was not cited in the article. The citation has now been inserted in the page 10, section **4 Discussion**, paragraph 5 and should read:

“Zhu, 2023b”

In the **Abstract**, The mistake was the non-uniform writing of “vs.”. This has been corrected to read: “The level of plasma *Aspergillus* IgG LFA in the IA group was significantly higher than that in the control group (190.5 AU/mL vs 50.3 AU/mL, P < 0.001)”

1. Page 06, section **3 Results**, sub-section 3.3, paragraph 2

The mistake was the non-uniform writing of “vs.”.

“Compared to the *Aspergillus* IgG ELISA with a cut-off value of 80 AU/mL, the *Aspergillus* IgG LFA had equivalent sensitivity (65.1% vs. 69.8% for sensitivity, P = 0.549), but significantly higher specificity (97.5% vs. 87.5% for specificity, P = 0.021)(Table 5).”

The correct statement is “vs”.

“Compared to the *Aspergillus* IgG ELISA with a cut-off value of 80 AU/mL, the *Aspergillus* IgG LFA had equivalent sensitivity (65.1% vs 69.8% for sensitivity, P = 0.549), but significantly higher specificity (97.5% vs 87.5% for specificity, P = 0.021)(Table 5).”

2. Page 06, section **3 Results**, sub-section 3.4

The mistakes were the non-uniform writings of “vs.” and “to”.

“The ROC curves of *Aspergillus* IgG LFA had no significant differences between patients with acute and subacute IA (AUC0.812[95% CI: 0.705, 0.919] vs 0.874 [95% CI: 0.780 to 0.967], P =0.401)(Figure 3D). The sensitivities, PPVs and NPVs of the *Aspergillus* IgG LFA were equivalent between patients with acute and subacute IA (57.6% vs. 73.3% for sensitivity, P = 0.190; 90.5% vs. 91.7% for PPV, P = 1.000; 84.8% vs. 90.7% for NPV, P =0.231)(Table 5).”

The correct statements are “vs” and “,”.

“The ROC curves of *Aspergillus* IgG LFA had no significant differences between patients with acute and subacute IA (AUC0.812[95% CI: 0.705, 0.919] vs 0.874 [95% CI: 0.780, 0.967], P =0.401)(Figure 3D). The sensitivities, PPVs and NPVs of the *Aspergillus* IgG LFA were equivalent between patients with acute and subacute IA (57.6% vs 73.3% for sensitivity, P = 0.190; 90.5% vs 91.7% for PPV, P = 1.000; 84.8% vs 90.7% for NPV, P =0.231)(Table 5).”

3. Page 09, section **4 Discussion**, paragraph 2

The mistake was the wrong writing of “65.1%”.

“In this study, the sensitivity and specificity of the BALF GM were 65.1% and 90.0%, respectively (Table 6).”

The correct statement is “65.0%”.

“In this study, the sensitivity and specificity of the BALF GM were 65.0% and 90.0%, respectively (Table 6).”

4. Page 09, section **4 Discussion**, paragraph 2

The mistakes were the non-uniform writings of “vs.” and “*P*”.

“A meta-analysis showed that PCR had higher sensitivity for the diagnosis of IA in non-neutropenic patients such as COPD, solid tumors and autoimmune diseases with prolonged corticosteroid therapy, compared to those with HM and/or HSCT/SOT (88% vs. 68%, *P* < 0.001) (Han et al., 2023).”

The correct statements are “vs” and “P”.

“A meta-analysis showed that PCR had higher sensitivity for the diagnosis of IA in non-neutropenic patients such as COPD, solid tumors and autoimmune diseases with prolonged corticosteroid therapy, compared to those with HM and/or HSCT/SOT (88% vs 68%, P < 0.001) (Han et al., 2023).”

